# Abdominal Aortic Access for Thoracic Endovascular Aortic Repair: A Safe and Effective Alternative in Challenging Vascular Anatomy

**DOI:** 10.7759/cureus.77887

**Published:** 2025-01-23

**Authors:** Kokoro Tabata, Shunya Ono, Motoharu Shimozawa, Kosaku Nishigawa, Takeyuki Kanemura

**Affiliations:** 1 Cardiovascular Surgery, IMS Katsushika Heart Center, Tokyo, JPN

**Keywords:** abdominal aortic approach, alternative treatments, complex vascular anatomy, descending thoracic aortic aneurysm, thoracic endovascular aortic repair

## Abstract

Thoracic endovascular aortic repair (TEVAR) is a minimally invasive treatment for descending thoracic aortic aneurysms, especially in high-risk patients; however, vascular access can be difficult in cases with small or calcified femoral arteries. Herein, we report a case of an 82-year-old woman with a descending thoracic aortic aneurysm who underwent TEVAR using an abdominal aortic approach because of inadequate femoral access. A synthetic graft was anastomosed to the abdominal aorta to provide a secure conduit for the deployment of the stent graft. The procedure was successfully completed without complications, and follow-up imaging revealed no endoleak or enlargement of the aneurysm. This case underscores the abdominal aortic approach as a safe and effective alternative for TEVAR in patients with complex vascular anatomy, offering favorable outcomes with technical refinements, such as placement of the synthetic graft and double tourniquets to minimize bleeding.

## Introduction

Descending thoracic aortic aneurysms pose a substantial risk of rupture and present complex management difficulties. In the treatment of descending thoracic aortic aneurysms, thoracic endovascular aortic repair (TEVAR) is considered advantageous compared to left thoracotomy in terms of perioperative mortality and complication rates, making it a particularly suitable option for elderly patients and those with comorbidities [1-3]. Although the femoral artery is generally used for vascular access, severe calcification or an inadequate vessel diameter may necessitate alternative routes [4,5]. Herein, we report a case of TEVAR conducted via the abdominal aorta, which provides a safe and minimally invasive solution for difficult femoral access.

## Case presentation

The patient, an 82-year-old woman, was referred to our institution following an incidental diagnosis of a descending thoracic aortic aneurysm during a routine health checkup that included a plain chest computed tomography (CT). The patient had no significant medical history, including hypertension, diabetes, or hyperlipidemia, and no family history of cardiovascular or aortic diseases. The patient had a longstanding smoking habit of 20 cigarettes per day for over 50 years, and CT imaging revealed emphysema. Preoperative assessment classified her as American Society of Anesthesiologists Physical Status (ASA-PS) class Ⅱ, primarily due to her emphysema. Additionally, her functional status was evaluated as New York Heart Association (NYHA) class I, reflecting no limitations in ordinary physical activities.

**Figure 1:**
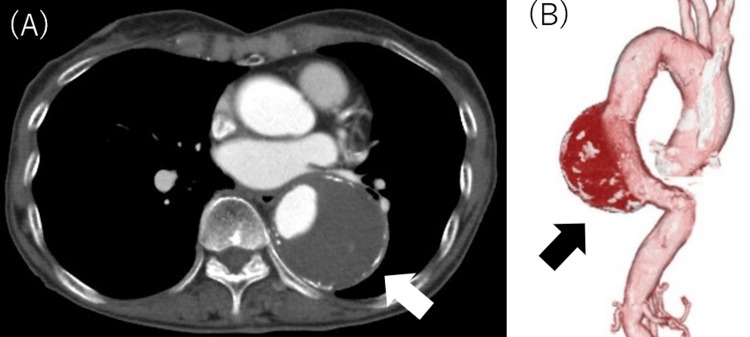
Preoperative contrast-enhanced CT. (A) Short-axis image revealing a 60 × 52 mm aneurysm with an abundant mural thrombus (white arrow). (B) A 3D reconstructed image revealing an aneurysm in the descending thoracic aorta in the region from Th8 to Th11 (black arrow). The image is visualized from a dorsal perspective.

The plain chest CT revealed a suspicious lesion, which was further evaluated using contrast-enhanced CT (CE-CT), confirming a descending thoracic aortic aneurysm measuring 60 × 52 mm, located between the levels of Th8 and Th11, without evidence of rupture or dissection. The aneurysm was saccular in shape, and no ulcerations were observed (Figure 1).

The descending thoracic aortic aneurysm had a maximum short-axis diameter exceeding 50 mm, and surgical intervention was planned in accordance with the Japanese guidelines for the diagnosis and treatment of aortic aneurysm and aortic dissection (Japanese Circulation Society/Japanese Society for Cardiovascular Surgery/Japanese Association for Thoracic Surgery/Japanese Society for Vascular Surgery 2020) [6]. Considering the patient's advanced age, the presence of emphysema as a comorbidity, and the assessment that an appropriate landing zone could be secured for TEVAR, we opted for TEVAR instead of left thoracotomy.

An 8-mm sheath insertion was deemed necessary for stent graft deployment; however, the patient’s femoral and iliac arteries had diameters of approximately 5 mm, making the standard femoral artery approach difficult. Therefore, we chose the abdominal aortic approach.

**Figure 2:**
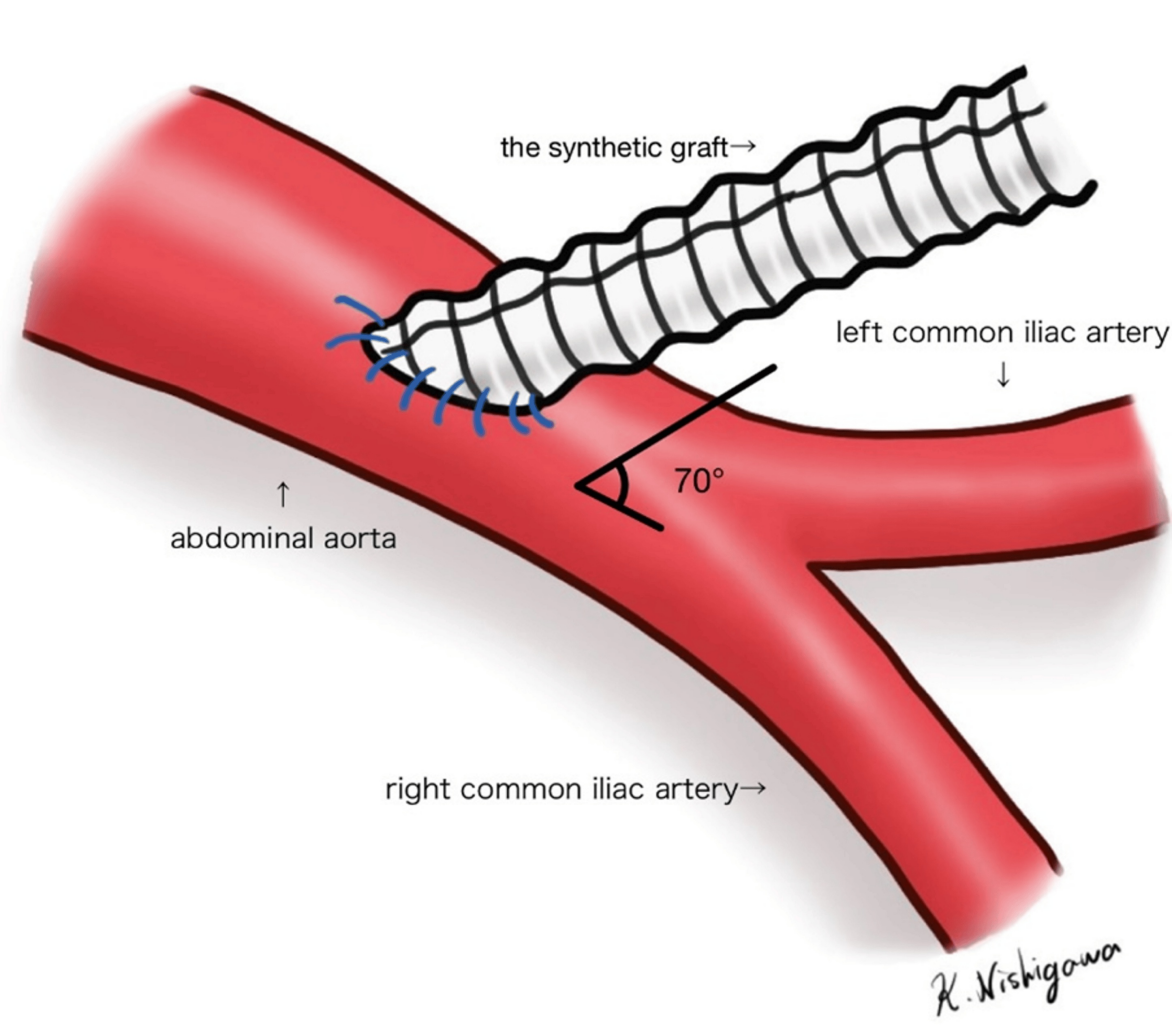
A synthetic graft anastomosis. The graft was cut at a 20° angle and anastomosed at a 70° caudal tilt. Image credits: Kosaku Nishigawa.

Surgery was initiated in the supine position under general anesthesia. Through a 7-cm midline laparotomy, a synthetic graft (10 × 300 mm, Triplex Straight, Terumo, Tokyo, Japan) cut at a 20° angle with a caudal tilt was sutured end-to-side to the abdominal aorta using 4-0 polypropylene continuous sutures, establishing a secure and sufficiently large conduit for introducing the stent grafts (Figure 2). The anastomosis site of the synthetic graft was at the L4 level. Using a Satinsky clamp during anastomosis of the synthetic graft effectively prevented backflow from the lumbar arteries, providing a clear surgical field.

**Figure 3:**
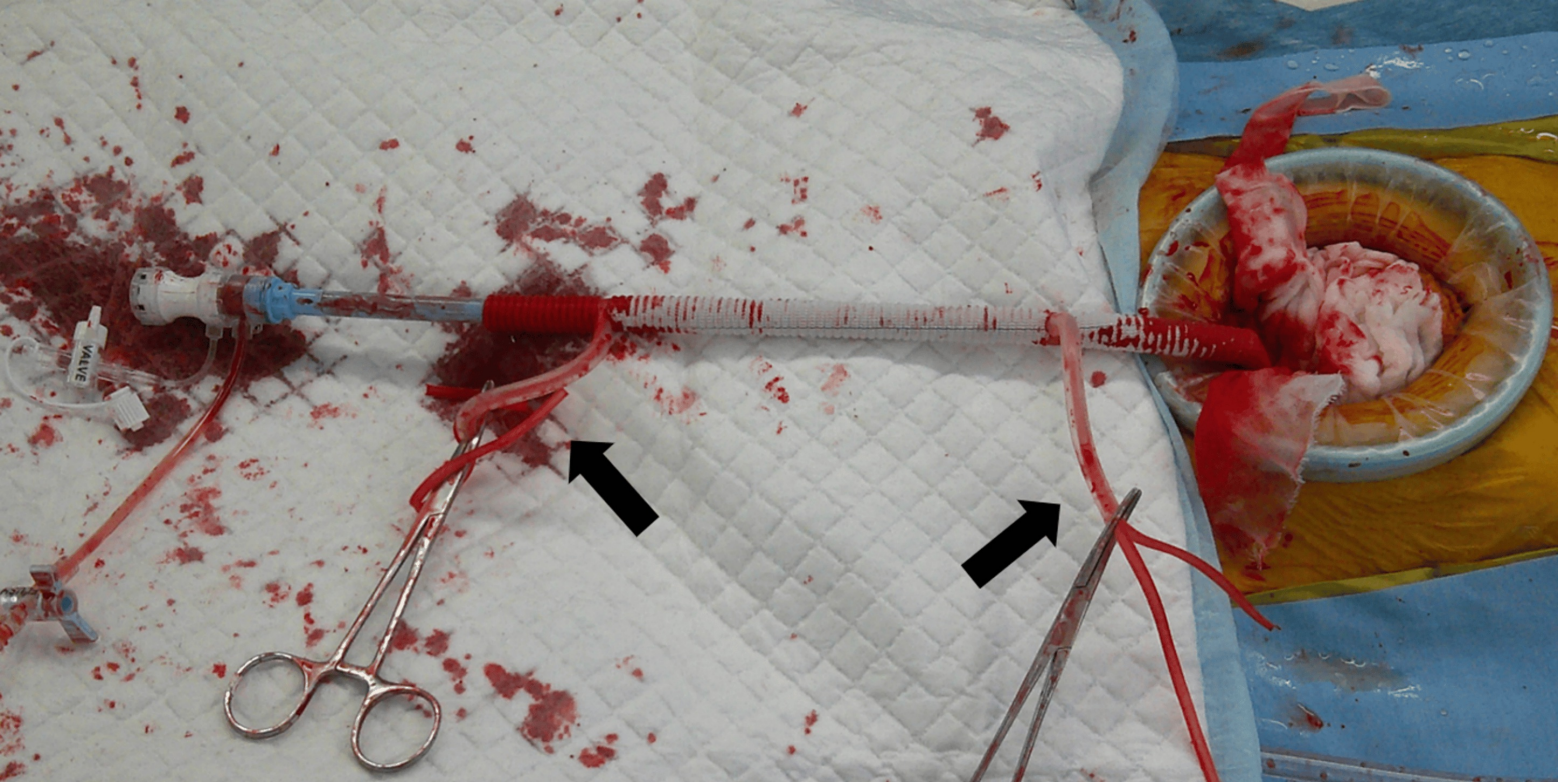
Intraoperative findings. The anastomosed synthetic graft was routed outside the abdominal cavity and the sheath was placed within the graft. Double tourniquets (black arrows) were used to prevent bleeding from the graft. The top image represents the operator’s side, the right side represents the patient’s cranial side, and the left side represents the patient’s caudal side.

An 8 mm DrySeal Flex Introducer Sheath (W. L. Gore & Associates, Flagstaff, AZ) was inserted through the synthetic graft and secured using double tourniquets (Figure 3). The first stent graft (tapered 30-26 mm × 150 mm, Valiant Captivia, Medtronic, Santa Rosa, CA) was deployed proximal to the celiac artery bifurcation. The second proximal stent graft (straight 32 × 150 mm, Valiant Captivia) was inserted into the first open stent and deployed just distal to the left subclavian artery bifurcation. Final angiography confirmed proper positioning, with no endoleak and adequate landing zones of more than 40 mm securely achieved (Video 1). After completing the angiography, the synthetic graft was ligated at its base using 2-0 silk sutures, the stump was closed with continuous suturing using 2-0 braided polyester sutures, transected, and the abdominal cavity was closed. The patient was extubated in the operating room and transferred to the recovery ward in stable condition.

**Video 1:** Intraoperative final angiography. No endoleak was observed, and the left subclavian artery remained patent.

**Figure 4:**
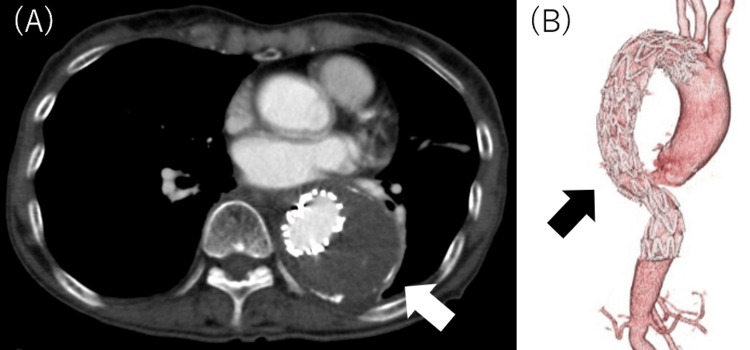
Postoperative contrast-enhanced CT. (A) No endoleak was observed on the short-axis image (white arrow). (B) A 3D reconstructed image. The stent graft was deployed distal to the left subclavian artery bifurcation and proximal to the celiac artery bifurcation (black arrow). The image is visualized from a dorsal perspective.

Postoperative CE-CT on postoperative day (POD) five revealed no endoleak, stent migration, or stenosis of the left subclavian artery and celiac artery, and no evident embolism was observed (Figure 4). The patient’s recovery was uneventful. The patient maintained stable hemodynamics, good respiratory function, and no signs of graft-related complications. Additionally, there were no bowel complications or wound-related issues associated with the laparotomy performed during the procedure. Due to the favorable clinical course, the patient was discharged on POD seven in a general condition comparable to her preoperative status. At follow-up, the patient reported no issues and was able to maintain her preoperative level of daily activities without limitations. The patient continued to show a stable and uneventful recovery during outpatient visits.

## Discussion

This case demonstrates the efficacy of a hybrid approach using access through the abdominal aorta for treating descending thoracic aortic aneurysms in older patients in whom vascular access is difficult. In the treatment of descending thoracic aortic aneurysms, no randomized controlled trials (RCTs) comparing TEVAR and left thoracotomy have been conducted; however, comparisons based on case reports and observational studies have been performed. These studies suggest that TEVAR is advantageous over left thoracotomy in terms of perioperative mortality and complication rates, though there is ongoing debate regarding its long-term outcomes [1,2,7,8]. TEVAR is particularly well-suited for elderly patients and those with comorbidities such as emphysema [1,3]. In the present case, we chose to perform TEVAR. Considering the patient’s advanced age and considerable emphysematous alterations in the lungs, left thoracotomy was considered to pose a high complication risk. There are reports suggesting that TEVAR for straight aortic anatomy improves landing stability, making cases of descending thoracic aortic aneurysms, such as the present case, well-suited for TEVAR [9].

Successful TEVAR requires adequate vascular access, and the femoral artery is typically the preferred access route [4,5]. Herein, the femoral or iliac arterial diameter was approximately 5 mm, which made peripheral arterial access difficult. Therefore, we used an abdominal aortic approach. Other access routes such as the ascending aorta, carotid artery, and subclavian artery are sometimes used [10]. However, using the ascending aorta or common carotid artery poses a risk of cerebral infarction [11,12]. Furthermore, in the present case, the common carotid and subclavian arteries were unsuitable for access because their diameters were approximately 6 mm, making abdominal aortic access the most appropriate choice. The abdominal incision was approximately 7 cm and the surgery was completed in a relatively short duration (132 minutes). This shows that even in cases where access is challenging, choosing abdominal aortic access allows for a safe procedure while maintaining a minimally invasive approach.

Notably, a synthetic graft was used to access the abdominal aorta. Instead of directly puncturing the abdominal aorta, suturing a synthetic graft to the abdominal aorta facilitated a safer and more controlled access route. Direct puncture carries risks, including bleeding at the puncture site and injury to surrounding structures, and requires procedures within the abdominal cavity. Using a synthetic graft allowed for sheath placement and other procedures to be conducted outside the abdominal cavity, making the entire operation safer and more manageable. However, direct puncture may be considered an alternative approach in cases in which severe calcification of the abdominal aorta makes clamping challenging [13].

The synthetic graft was pre-cut at a 20° angle and anastomosed to the abdominal aorta with a caudal tilt (Figure 2). This aided in smoother insertion of the sheath and ensured easier alignment. Furthermore, the use of double tourniquets to control blood flow helped minimize intraoperative bleeding from the synthetic graft, thereby contributing to the overall success of the procedure (Figure 3). These technical refinements helped in ensuring successful outcomes.

Achieving vascular access is the main challenge in TEVAR. This case demonstrates that access to the abdominal aorta can be an effective alternative when femoral access is onerous. Although reports on TEVAR via abdominal aortic access are limited, further research is required to examine long-term outcomes and refine these techniques. Incorporating methods, such as synthetic graft anastomosis, into larger studies will possibly provide stronger evidence of their effectiveness and aid in preventing further complications.

## Conclusions

As treatment options for descending thoracic aortic aneurysms, both TEVAR and left thoracotomy are available. While there is ongoing debate regarding the long-term outcomes, TEVAR is particularly well-suited for elderly patients and those with comorbidities. This case shows that abdominal aortic access in TEVAR provides an effective alternative for patients in whom peripheral arterial access is not feasible. A safe and controlled access route can be established using a synthetic graft, which considerably improves the safety of the procedure. Furthermore, technical refinements, such as angled graft cuts and using double tourniquets, further minimize bleeding and reduce complication risks. TEVAR through abdominal aortic access offers a promising option for patients with complex vascular anatomy or comorbidities, providing a minimally invasive and effective treatment. Further research and clinical experience are expected to enhance these techniques and improve the outcomes for high-risk patients.
